# Molecular Evolution and Phylodynamics of Acute Hepatitis B Virus in Japan

**DOI:** 10.1371/journal.pone.0157103

**Published:** 2016-06-09

**Authors:** Serena Y. C. Lin, Hidenori Toyoda, Takashi Kumada, Hsin-Fu Liu

**Affiliations:** 1 Department of Bioscience and Biotechnology, National Taiwan Ocean University, Keelung, Taiwan; 2 Department of Medical Research, Mackay Memorial Hospital, Taipei, Taiwan; 3 Department of Gastroenterology, Ogaki Municipal Hospital, Ogaki, Gifu, Japan; 4 Department of Nursing, National Taipei University of Nursing and Health Sciences, Taipei, Taiwan; University of Cincinnati College of Medicine, UNITED STATES

## Abstract

Hepatitis B virus (HBV) is prevalent worldwide and causes liver diseases, including acute and chronic hepatitis. Ten HBV genotypes (A–J) with distinct geographic distributions have been reported. Cases of acute HBV infection with genotype A have increased in Japan nationwide since the 1990s, mainly through sexual transmission. To investigate the molecular evolution and phylodynamics of HBV genotypes, we collected acute HBV isolates acquired in Japan from 1992–2002. Full genomes were obtained for comprehensive phylogenetic and phylodynamic analysis, with other Japanese HBV sequences from GenBank that were isolated during 1991–2010. HBV genotypes were classified using the maximum-likelihood and Bayesian methods. The GMRF Bayesian Skyride was used to estimate the evolution and population dynamics of HBV. Four HBV genotypes (A, B, C, and H) were identified, of which C was the major genotype. The phylodynamic results indicated an exponential growth between the 1960s and early 1990s; this was followed by a population bottleneck after 1995, possibly linked with successful implementation of a nationwide vaccination program. However, HBV/A increased from 1990 to 2003–2004, and then started to decrease. The prevalence of genotype A has increased over the past 10 years. Phylodynamic inference clearly demonstrates a steady population growth compatible with an ongoing subepidemic; this might be due to the loss of immunity to HBV in adolescents and people being born before the vaccination program. This is the first phylodynamic study of HBV infection in Japan and will facilitate understanding the molecular epidemiology and long-term evolutionary dynamics of this virus in Japan.

## Introduction

Hepatitis B virus (HBV) is a circular, partially double-stranded DNA virus belonging to the *Hepadnaviridae*, and is one of the major causes of liver diseases, including acute and chronic hepatitis, that lead to liver cirrhosis or hepatocellular carcinoma (HCC). HBV is highly prevalent in South and East Asia, especially in China, Japan, and Taiwan. Ten HBV genotypes (A–J) have been identified [1–4], showing pairwise differences from 8% to 17%. Genotype A is prevalent in Africa, North America, and Europe; genotypes B and C in Asia; E in Africa; F and H in Central and South America; I in Taiwan; and J in Japan. Genotypes D and G appear prevalent worldwide [2]. Different genotypes tend to have distinct geographic distributions and their clinical manifestations may differ. In East Asia, genotype C constitutes almost 100% of the infections in South Korea [5], approximately 50% in Hong Kong [6], and 85% in Japan [7]. In China, genotype C is predominant in the northern regions (e.g., Beijing, Xinjiang, and Gansu), whereas genotype B is prevalent in the central and eastern regions (e.g., Hunan and Fujian), with an overall prevalence of 41% for genotype B and 53% for genotype C [8]. However, in Taiwan, HBV genotype B is more prevalent (68%) than genotype C (32%) [9].

HBV genotypes are crucial in determining the clinical severity of HBV, hepatitis B e antigen (HBeAg) seroconversion, response to treatment of HBV infection, and possible vaccination against the virus [10, 11]. Genotyping of HBV is crucial to understanding the pathogenesis of the virus [12]. Genetic variants may differ in their patterns of disease outcome, virulence, and response to therapy. Additionally, HBV genotypes play a role in the development of HCC. The data from Taiwan showed that genotype C is associated with more severe liver disease, including cirrhosis, and a higher prevalence in younger HCC patients [13, 14], whereas genotype B is associated with the development of HCC in young noncirrhotic patients [15]. Previous reports have indicated that genotype C is the major genotype in chronic hepatitis, liver cirrhosis and HCC. Genotype C is associated with more severe liver disease than the B variants, whereas genotype B is more prevalent in acute liver disease than in chronic forms [16].

Hepatitis B surface antigen (HBsAg), or S protein, is abundant in virions and patients’ serum or plasma, and is encoded by the S gene. The core protein (HBcAg) is a polypeptide with a molecular size of 22 kDa that is encoded by the C gene of HBV. The precore sequence within the C gene is essential for the expression and secretion of HBeAg. It is a reliable marker for the presence of intact virions and infectivity. Spontaneous HBeAg loss during the course of chronic HBV infection is generally accompanied by a decrease in the serum HBV DNA level and viral replication [17, 18]. This process is known as HBeAg seroconversion. A336C/A336T/T337C mutations in the HBV pre-core gene were shown to cause HBeAg seroconversion in chronic patients by damaging the cleavage site of *Tsp*509I [17]. Moreover, A336C/A336T caused the substitution of Glu-83 with Asp in HBcAg [18]. Recent studies have indicated that HBeAg upregulation reduced the TLR level, which protected HBV from the immune response and led to a higher HBV DNA viral load in HBeAg positive patients [19, 20]. Therefore, it is reasonable to suspect that pre-core reduces serum HBV DNA and elevates the ALT level by downregulating HBeAg expression or promoting HBeAg loss [21].

According to an HBV genotyping study on patients with chronic hepatitis B in Japan in 2001, genotype C was the most prevalent (84.7%), followed by genotypes B (12.2%) and A (1.7%) [7]. However, genotype distribution differs considerably between chronic and acute hepatitis B groups. By contrast, genotype A (HBV/A) is frequently noted in acute hepatitis B patients [22]. Acute infection with HBV/A is increasing mainly through sexual contact and tends to persist longer than that with HBV genotype C [22–24].

The evolutionary dynamics of viruses may vary considerably depending on the type of transmission (e.g., vertical vs. intravenous injection) [25]. Therefore, investigating the phylodynamic pattern of HBV in Japan is worthwhile. Full HBV genomes were used to perform a comprehensive phylogenetic and evolutionary dynamic analysis. HBsAg has been identified as the major target of antibody neutralization. Hence, the selection pressure on the S gene was investigated. HBeAg seroconversion-related precore gene mutations also prompted investigation of the selection pressure on the C gene. This is the first phylodynamic study of HBV in Japan for a time span of 19 years. The findings of this study will provide essential information on the molecular epidemiology and long-term evolutionary dynamics of this virus in Japan.

## Material and Methods

### Patient samples

Plasma samples of the 57 patients with acute hepatitis B during 1992–2002 were collected from Ogaki Municipal Hospital, Japan. The study protocol was approved by the ethics committee of Ogaki Municipal Hospital in accordance with the 1975 Helsinki Declaration. Signed informed consent was obtained from each patient.

### HBV DNA extraction, PCR, and sequencing

Plasma samples were stored in a refrigerator at -80°C. To extract the HBV DNA, 200 μL of plasma was used by the High Pure Viral Nucleic Acid Kit and eluted in 50 μL of elution buffer (Roche, Cat. No. 11 858 874 001). The S and C genes of HBV were amplified with three primer pairs designed from conserved genome regions.

Five microliters of HBV DNA was added to a mixture containing 5 μL of 10x Ex Taq buffer, 4 μL of 2.5 mM dNTP mixture, 1 μL 10 μM sense primer, 1 μL 10 μM antisense primer, 2 units of Ex Taq polymerase (TaKaRa), and 33.5 μL of d.d. H_2_O. Primers S1 and S2 were used for the S gene amplification [26]. Primers x1281F (TCGGCGCTGAATCC), s37R (CCGCCTGTAACACGAG), ChF2 (TACTAGGAGGCTGTAGGCATA), and ChR2 (ATCTCGAATAGAAGGAAAGAAGTC) were used for the C gene amplification. PCR for the S gene was performed under the following cycling conditions: denaturation at 95°Cfor 2.5 min; 45 cycles at 95°Cfor 30 sec and 55°C for 30 sec and extension at 72°C for 45sec; and a final elongation at 72°C for 7 min. PCR for the C gene was performed under the following cycling conditions: denaturation at 95°C for 2 min; 45 cycles at 95°C for 30 sec and 55°C for 30 sec and extension at 72°C for 90 sec; and a final elongation at 72°C for 7 min.

Full HBV genome sequences were used for the phylodynamic analysis. Full-length HBV genomes were amplified using nested PCR. The first round of PCR was performed with a single set of primers: sense primer P1 (5_-CCGGAAAGCTTGAGCTCTTCTTTTTCACCTCTGC CTAATCA-3_ 1821–1841) and antisense primer P2 (5_-CCGGAAAGCTTGAGCTCTTCAAAAAGTTGCATGGTGCTGG-3_ 1825–1806). The second round of PCR was performed with a different primer pair: sense primer P3 and antisense primer P4, as described by Gunther et al. [27]. The PCR amplification program followed the prescription of Chen et al. [28].

The PCR product was confirmed through agarose-gel electrophoresis by using 2% agarose gel with ethidium bromide staining and UV transillumination. Subsequently, the PCR product was purified using an illustra^™^ GFX^™^ PCR DNA and Gel Band Purification Kit (GE). The purified DNA was direct-sequenced with primers HS1, HS2, x1281F, s37R, ChF2, ChR2, HBVc1F (ACTTCCGGAAACTACTGTT), and HBVc2R (GAGATTGAGATCTTCTGCGA) by using the Big Dye Terminator v3.1 Cycle Sequencing Kit (Applied Biosystems). The sequencing primers for the full genome are shown in S1 Table. Five microliters of template DNA was added to the master mix containing 1 μL of Big Dye, 4 μL of Big Dye buffer, 2μL of 1 μM sequencing primer, and 8 μL of dd H_2_O. The Big Dye reaction was carried out using 25 cycles at 96°C for 10 s, 50°C for 5 s, and 60°C for 4 min. The sequencing samples were purified using the illustra Sephadex^™^ G-50 (GE) and then sequenced using the ABI 3730 DNA Analyzer (Applied Biosystems).

### Data mining of HBV DNA sequences

Viral nucleotide sequences isolated from acute hepatitis B patients were confirmed by Nucleotide BLAST on NCBI. The S and C genes of the 57 samples and other HBV reference strains, including genotypes A–J and nonhuman HBV (gibbon) sequences from the GenBank database, were used for genotyping analysis.

The full genome of Japanese HBV and the acute HBV sequences mined from GenBank and from 1991–2010 were collected to perform a phylodynamic analysis. Other full genome HBV datasets focusing on genotype A (HBV/A) in Japan and from 1993–2009 were collected. The accession numbers of the HBV sequences included in all analyses are listed in S2 Table.

### Genotyping

Multiple sequence alignment was performed using the MUSCLE [29, 30] program with the MEGA 5 package [31]. Phylogenetic tree reconstruction and statistical evaluation were performed using the BEAST v1.7.4 [32] package for the Bayesian inference, and MEGA 5 for the ML method with bootstrap analysis (1000 replicates). The nearest-neighbor-interchange (NNI) method was employed for searching the heuristic ML tree. All HBV sequences were aligned and a jModelTest was used to determine the best-fitting nucleotide substitution model [33] (http://darwin.uvigo.es/our-software/). The best-fitting model for the HBV S and C genes was a general time-reversible model with a discrete gamma distribution (+G) of five rate categories, with the assumption that a certain fraction of sites are evolutionarily invariable (+I). Possible recombination events were analyzed using Simplot software [34] (http://sray.med.som.jhmi.edu/SCRoftware/simplot/). The HBV genotyping was confirmed using the online tool BioAfrica (http://bioafrica.mrc.ac.za/).

### Phylodynamics and evolutionary rate

To investigate the viral population dynamics and evolutionary rate of HBV, we used the BEAST program to implement the coalescent-based Bayesian Markov Chain Monte Carlo (MCMC) method. Statistical support for monophyletic clades was assessed by calculating the posterior probability, with p>0.95 indicating significant support. In addition, we compared the parametric constant and exponential growth demographic models with the nonparametric Bayesian skyline plot (BSP) [35] and Gaussian Markov random field (GMRF) Skyride plot [36] models under both strict and relaxed clock combinations, with lognormal distribution rates. After discarding the first 10% of the MCMC samples, proper mixing of the Markov Chain was assessed by calculating the effective sampling size (ESS>200, suggesting proper mixing) by using Tracer v1.4 software (http://tree.bio.ed.ac.uk/software/tracer/),. Uncertainty estimates were quantified by 95% highest posterior density (95% HPD) intervals. The tree with the maximum sum of posterior probabilities (maximum clade credibility) and a 10% burn-in was obtained from the posterior distribution with Tree Annotator v1.5.4 and observed using FigTree v1.3.1.

### Selection pressure on HBV genes

When viruses undergo natural selection, evolution occurs by the random processes of mutation. The rate at which amino-acid altering (nonsynonymous) substitution occurs (*d*N) in the codon is compared with the rate of nonaltering (synonymous) substitutions (*d*S). When amino-acid-altering substitutions are disadvantageous, the value of the *d*N/*d*S ratio is negative. Contrastingly, the value of the *d*N/*d*S ratio would be positive if the substitutions are beneficial. Selection pressure acting on the HBV gene was investigated using the fixed effects likelihood (FEL) method in the HYPHY [37] package on the Datamonkey Web-based interface (http://www.datamonkey.org).

## Results

### Genotyping

**Fig 1:**
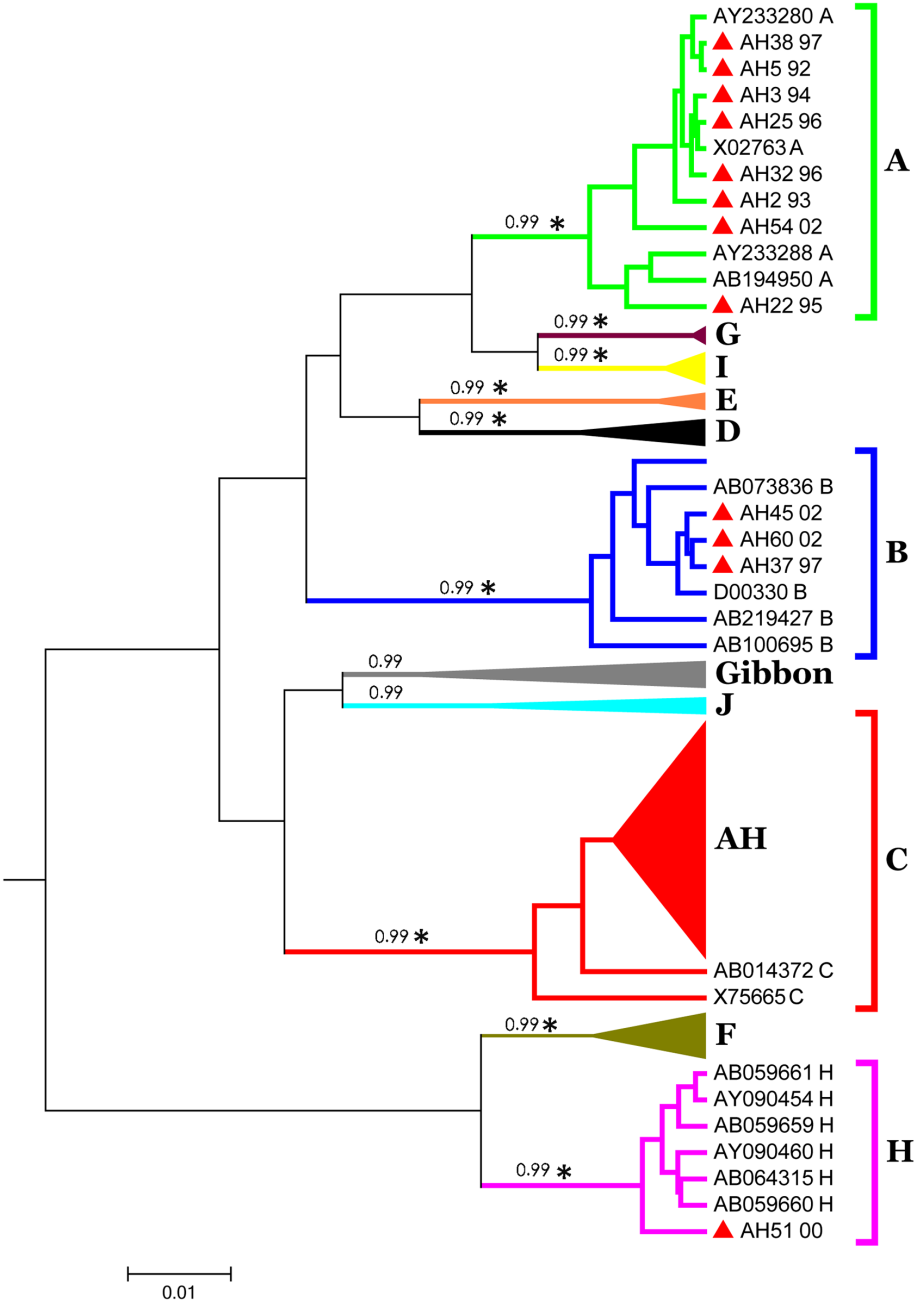
Phylogenetic tree of HBV S gene reconstructed by Bayesian inference. The posterior probabilities higher than 0.95 and the star (* = bootstrap value greater than 75) on the branches indicate a significant cluster. Red triangles present acute hepatitis (AH) samples obtained from Japan (n = 57) were divided into genotype A, B, C and H.

The S and pre-C/C genes were direct-sequenced from the PCR product after purification. These Japanese acute HBV sequences were submitted to NCBI GenBank and assigned accession numbers KC836778–KC836829 (S gene), KC836830–KC836876 (pre-C/C gene), and KC836877–KC836881 (full genome). Our acute hepatitis B samples were classified into four genotypes. A consistent tree topology was obtained using the ML and Bayesian methods (300 million MCMC strict-constant prior). Of the 57 plasma samples, 14% (n = 8) are genotype A; 5% (n = 3) are genotype B; 78% (n = 45) are genotype C; and only one case (1%) is genotype H, identified according to the S gene (Fig 1).

**Fig 2:**
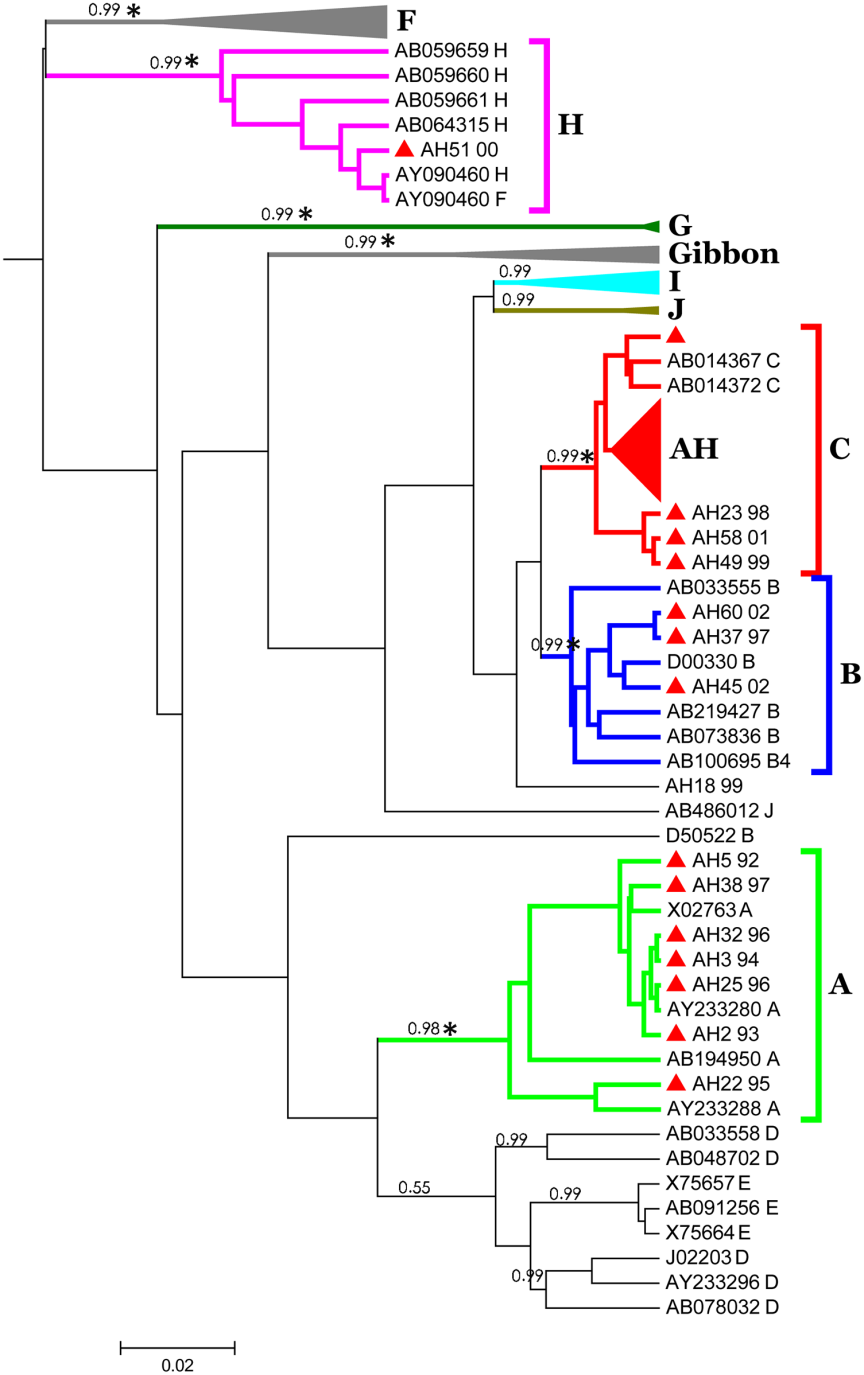
Phylogenetic tree of HBV C gene reconstructed by Bayesian inference. The posterior probabilities higher than 0.95 and the star (* = bootstrap value great than 75) on the branches indicate a significant cluster. Red triangles present samples obtained from Japan (n = 48) were divided into genotype A, B, C and H.

Only 46 of the 57 samples of the pre-C/C gene were successfully PCR-amplified and sequenced. The other 11 samples consistently failed on sequencing, despite the sequencing primers and conditions being the same as those for the 46 successful samples. Other sequencing primers designed for the adjacent regions also failed to sequence out these samples. Of the 46 pre-C/C sequences, 14% (n = 7) of the samples are genotype A; 6% (n = 3) are genotype B; 73% (n = 35) are genotype C; and 1% (n = 1) is genotype H (Fig 2). Genotyping of the two genes confirmed that genotype C is the most prevalent type. Analysis with Simplot software detected no recombination event in these isolates.

### Phylodynamics

**Fig 3:**
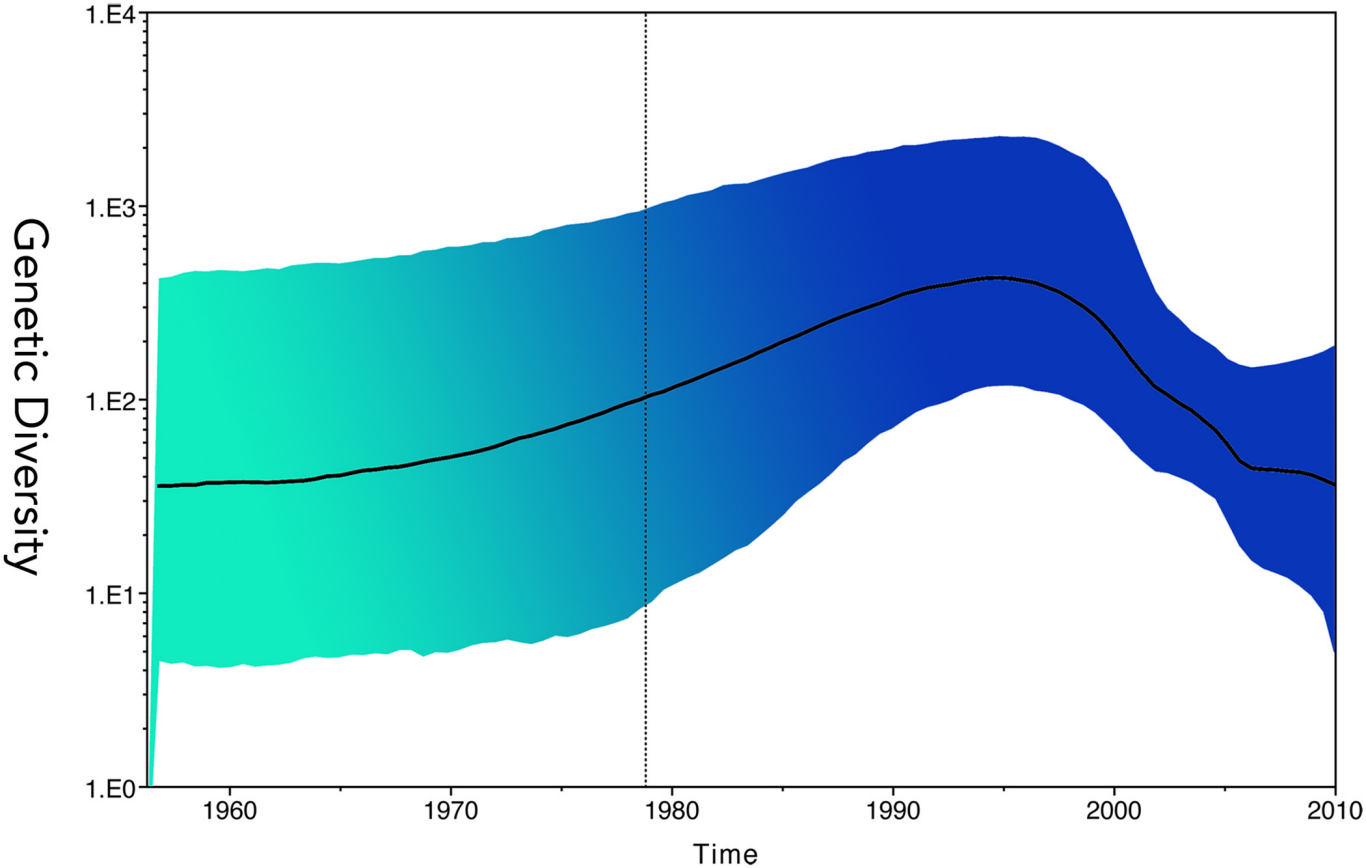
Phylodynamic analysis of HBV in Japan. Full genome of acute hepatitis B sequences and other Japanese HBV sequences from GenBank were included for GMRF Skyride analysis. The solid black line indicates the mean effective viral population size, as well, the genetic diversity. The genetic diversity of HBV in Japan has subsided since 1995. This change indicated that the virus was been through a bottleneck and agreed by both Upper and Lower HPD 95% curve.

Phylodynamic analysis of full-length sequences of all genotypes (1991–2010) of Japanese HBV by using the relaxed Skyride model (700 million MCMC) showed that the overall effective population size—including genotypes A, B, C, and others—grew exponentially between the mid-1960s and the early 1990s. Subsequently, after 1995, an obvious bottleneck effect occurred in the population (Fig 3).

**Fig 4:**
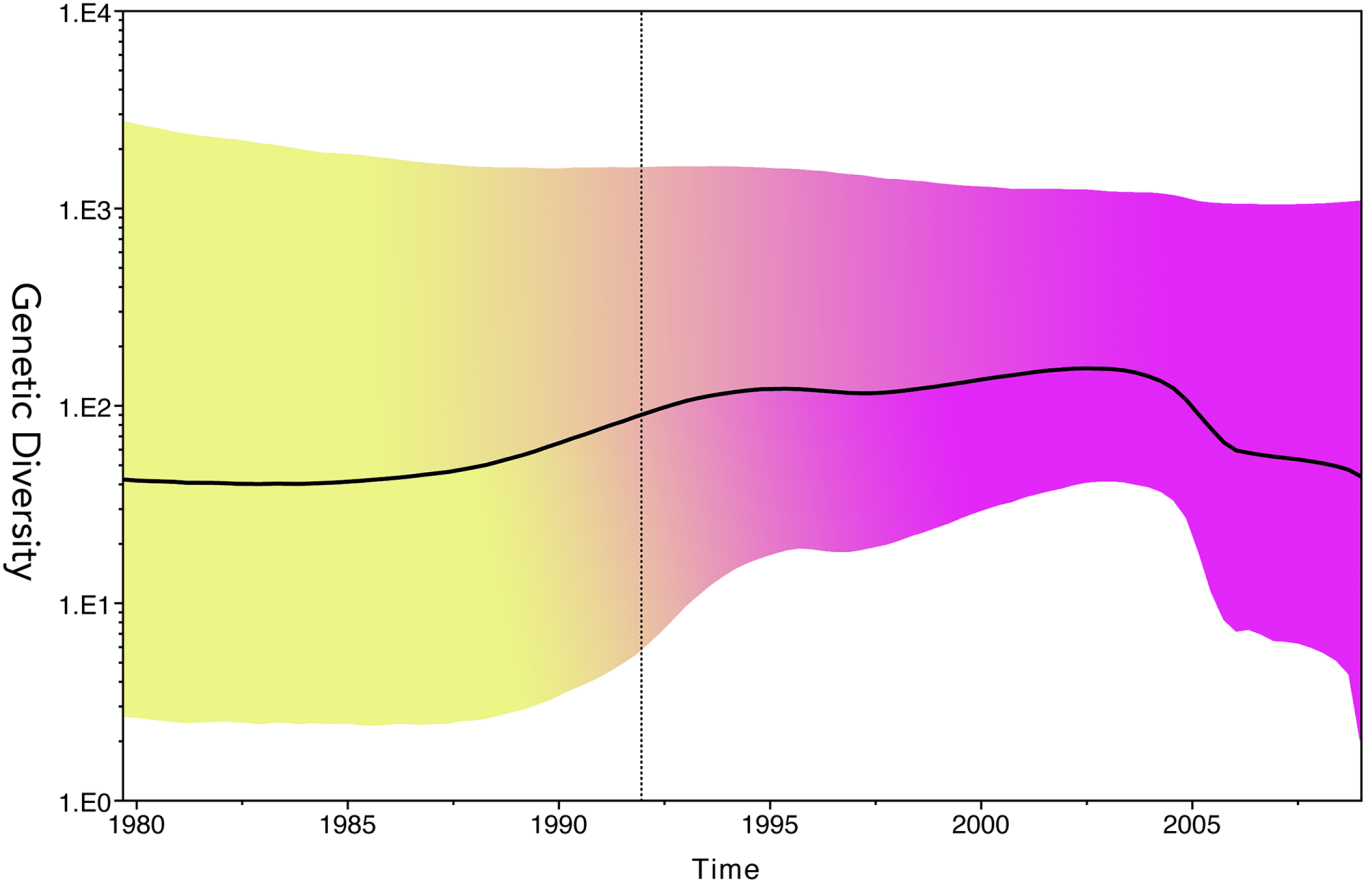
Phylodynamic analysis of genotype A in Japan. Full genomes of acute HBV genotype A samples and other Japanese HBV genotype A sequences retrieved from GenBank were used to run Skyride analysis. The Upper HPD 95% shows stable population maintaining over time while the Lower HPD 95% of effective number of viral population suggests exponential growing from early 1990 and a quick decline after 2003. However, the mean of genetic diversity (the solid black line) revealed that HBV/A population is substantially increasing from 1990 to 2003 and decreased since 2005.

However, the phylodynamic analysis of HBV/A indicated a different evolutionary pattern. The viral population increased from 1990 to 2003–2004 and then started to decrease (Fig 4).

### Evolutionary rates

The marginal likelihood indicated that the relaxed BSP was effective when it reached 900 million MCMC for both the S and C genes. The mean evolution rate of the S gene is 8.40E-5, (95% HPD: 1.35E-6–1.94E-4) and that of the C gene is 2.41E-3, 95% HPD: 1.56E-3–3.38E-3 subs/site/year. The C gene evolution rate is slightly higher than that of the S gene.

The evolution rate of the first and second codon positions (mean relative substitution rate 0.79, 95% HPD: 0.28–0.49) was significantly lower than that observed at the third codon position (mean relative substitution rate 1.42, 95% HPD: 1.11–1.77) of the S gene. This pattern was more pronounced for the C gene, where the evolution rate of the first and second codon positions (mean relative substitution rate 0.38, 95% HPD: 0.27–0.48) was significantly lower than that of the third codon position (mean relative substitution rate 2.24, 95% HPD: 2.03–2.46) (Tables 1 and 2). The mean evolution rate of HBV/A is 6.3E-4 (95% HPD: 2.5E-4–9.9E-4) after 700 million steps of MCMC.

**Table 1:** Mean relative evolutionary rates for codon positions in S genes of HBV.

	Mean relative substitution rate	SE of mean
S gene	8.40E-5(1.35E-6~1.94E-4)	
1^st^ + 2^nd^ codon position (95% HPD)	0.79 (0.62–0.94)	1.23E-3
3^rd^ codon position (95% HPD)	1.42 (1.11–1.77)	2.46E-3

SE: standard error

**Table 2:** Mean relative evolutionary rates for codon positions in C genes of HBV.

	Mean relative substitution rate	SE of mean
C gene	2.41E-3(1.55E-3~3.38E-3)	
1^st^ + 2^nd^ codon position (95% HPD)	0.38 (0.27–0.48)	7.76E-4
3^rd^ codon position (95% HPD)	2.24 (2.03–2.46)	1.55E-3

SE: standard error

### Selection pressures on the S gene and the C gene

The selection pressures on the S gene were estimated on the basis of the *d*N/*d*S ratio. FEL analysis showed two positively selected sites and 22 negatively selected sites (Table 3). For the C gene, FEL analysis showed one positively selected site and 22 negatively selected sites (Table 4).

**Table 3:** Selection sites in the S gene of acute hepatitis B viruses at 90% significance level.

Codon	Normalized *d*N-*d*S	P-value
45	25.31	0.04
184	26.60	0.04
71	-25.14	0.04
73	-44.28	0.003
82	-37.49	0.01
99	-24.18	0.05
100	-45.63	0.07
115	-26.93	0.02
117	-58.33	0.01
118	-32.20	0.2
120	-19.66	0.03
122	-56.62	0.004
125	-45.5	0.003
130	-20.37	0.07
136	-26.93	0.03
137	-21.01	0.09
144	-58.37	0.004
146	-68.66	0.002
154	-21.18	0.06
155	-80.50	0.0004
171	-23.58	0.034
190	-49.34	0.04
214	-31.29	0.02
215	-20.87	0.05

d_N_: non-synonymous sites; d_S_: synonymous sites

**Table 4:** Selection sites in the C gene of acute hepatitis B viruses at 90% significance level.

Codon	Normalized *d*N-*d*S	P-value
43	48.71	0.0003
58	-11.32	0.04
63	-11.23	0.05
69	-18.88	0.01
71	-11.35	0.07
72	-23.14	0.003
73	-16.85	0.01
94	-11.32	0.04
101	-12.51	0.03
114	-11.32	0.04
119	-11.23	0.05
123	-8.34	0.09
131	-11.23	0.05
133	-11.23	0.05
138	-12.39	0.03
148	-11.23	0.06
156	-15.10	0.01
158	-11.32	0.05
161	-15.10	0.01
163	-11.23	0.05
171	-13.21	0.05
172	-11.32	0.04
210	-13.82	0.02

d_N_: non-synonymous sites; d_S_: synonymous sites

## Discussion

The prevalences of A, B, C, D, and mixed genotypes in HBV in Japan were found to be 1.7%, 12.2%, 84.7%, 0.7%, and 1%, respectively (total n = 720) [7]. The distributions of genotypes A, B, C, and H were 14%, 5%, 78% and 1%, respectively (total n = 57). Our results accord with previous findings that indicate that genotype C is the predominant HBV genotype, and that the prevalence of HBV/A has increased over the past 10 years. When HBV/A was introduced to Japan from foreign countries in 1991, the incidence of acute infections increased rapidly and, subsequently, chronic infections increased [38]. Furthermore, an outbreak of HBV/A in patients coinfected with HIV-1 was reported in Japan [39]. Viral evolution dynamics may vary considerably between different types of transmission [25]; therefore, it is worthwhile to investigate the phylodynamic pattern of HBV genotypes in Japan. We searched all the Japanese full HBV/A genome sequences with known collection times that were available from GenBank and analyzed them together with our samples. To improve resolution, we used the GMRF Bayesian Skyride plot to investigate their population dynamics over time. Phylodynamic analysis showed that the overall effective population size of HBV, including genotype C, the most prevalent genotype, decreased after 1995; whereas HBV/A increased from 1990 to 2003–2004, and then started decreasing (Figs 3 and 4). The decrease in the overall effective population size of HBV in Japan has not been substantially reversed since the nationwide “mother-to-infant HBV infection prevention program” vaccination policy was launched in 1986 [40]. However, the increasing phylodynamic pattern of genotype A from 1990 to 2003–2004 was mainly a consequence of sexual transmission. This raises the question of whether hepatitis B vaccines have a less protective effect on HBV/A. Nevertheless, current recombinant HBV vaccines are of the A2 genotype, and all available data show that these vaccines are highly effective in preventing infections by all known HBV genotypes. Wu et al. reported that a significant proportion of complete vaccines may have lost their immunological memories against HBsAg in adolescents [41]. Furthermore, although the vaccination program has successfully protected most infants [42, 43], breakthrough infection cases after full HBV vaccination have been observed [44]. In Taiwan, children born from HBeAg-positive mothers are at a greater risk of chronic HBV infection (9.26%) despite immunization [45]. We propose that endemic Japanese HBV has been controlled by the nationwide vaccination program, as shown by the GMRF Bayesian Skyride plot. By contrast, the increasing phylodynamic pattern of HBV/A might be due to the loss of immunity to HBV in adolescents since HBV/A was introduced to Japan from foreign countries, mainly through sexual transmission. Antibody-naive adults older than 30 years who were born before the vaccination program are also considered potential factors for HBV/A population growth.

The HBV genotyping of surface and core genes are consistent in all the phylogenetic trees constructed by the ML and Bayesian methods. However, the HBV S gene has more favorable phylogeny and bootstrap support for monophyletic clades than the C gene does. For instance, typing by the phylogeny of the C gene revealed that the genotype C reference strain X75665 did not fall into the genotype C cluster, and neither did the genotype J strain AB486012 to the genotype J cluster. However, they were all properly classified into their appropriate cluster according to their genotype by using full genome sequences. The possibility of gene recombination was ruled out by Simplot analysis. Apparently the S gene is a more favorable HBV genotyping marker than the C gene and is suitable for fast screening.

According to Bayesian analysis, the evolution rate of the C gene is faster than that of the S gene (2.41E-3 vs. 8.40E-5 subs/site/year). Selection pressures estimated using the *d*N/*d*S ratio also indicated that the C gene has more selection sites than the S gene. All the results suggest that the C gene has a faster rate of genetic change. Similar results have been observed in chronically infected patients by Van de Klunder et al. who found that the nucleotide substitution rate of the HBV C gene was higher (8.15 × 10^−4^ vs. 4.57 × 10^−4^ subs/site/year) than that of the S gene [46].

A336C/A336T/T337C mutations in the C gene are HBeAg seroconversion related, so checking the selection pressure on different ORFs appeared relevant. However, no positive or negative selection site located in coding position 112 on the C gene was found. In a previous study, 19 positive selection sites in the C gene were reported in HBeAg negative chronic hepatitis B patients [47]. Six positive and 10 negative selection sites in the S gene reached statistical significance in chronic HBV sequences [48]. This high frequency of nonsynonymous mutations on the C gene was considered responsible for impaired antiviral immunity and the high levels of viral replication that cause liver inflammation in chronic hepatitis B. In this study, one codon in the C gene and two codons in the S gene were identified as being under positive selection pressure in acute hepatitis B. However, in chronic hepatitis B patient samples (data not shown), there are six positive selection sites in the S gene. This difference might be a consequence of HBV encountering disparate environmental pressures at different disease stages.

## Supporting Information

S1 Table Sequencing Primers for HBV full genome. (DOCX)

S2 Table Accession numbers of HBV datasets used in different analysis. (DOCX)
