# Supplementary material for: Molecular Evolution and Phylodynamics of Acute Hepatitis B Virus in Japan
Source: PLoS One. 2016 Jun 9;11(6):e0157103. doi: 10.1371/journal.pone.0157103 (PMC4900519; doi:10.1371/journal.pone.0157103)
Supplement: S1 Table — (DOCX) [file pone.0157103.s001.docx]

**S1 Table. Sequencing Primers for HBV full genome**

| ID |  | Primer Sequence |
| --- | --- | --- |
| P1 | F | 5_-CCGGAAAGCTTGAGCTCTTCTTTTTCACCTCTGC CTAATCA-3_ (1821–1841) |
| P2 | R | 5_-CCGGAAAGCTTGAGCTCTTCAAAAAGTTGCATGGTGCTGG-3_ (1825–1806) |
| P3 | F | 5-CTTTTTCACCTCTGCCTAATCATCTCATGTTCATGTCCTA-3_(1820–1859) |
| P4 | R | 5-TTGCATGGTGCTGGTGAACAGACCAATTTATGCCTACA-3_ (1819–1782) |
| F4 | F | 5-GTCACCATATTCTTGGGAAC-3_(2816-2835) |
| F6 | F | 5-CCAATTTGTCCTGGCTATCGC-3_(353-373) |
| 1718R | R | 5-ACAGTCTTTGAAGTAKGCCTCAAGG-3_(1718-1694) |
| 2299F | F | 5-CCACCAAATGCCCCTATCTTATCAAC-3_(2299-2325) |
| R4 | R | 5-GAGGACAAACGGGCAACA-3_(479-462) |
| S1 | F | 5-TGGCTCCAGTTCAGGAACAG-3_(67-86) |
| S483F | F | 5-TTAGGGTTTAAATGTATACCCA-3_(668-683) |
