# Supplementary material for: Molecular Evolution and Phylodynamics of Acute Hepatitis B Virus in Japan
Source: PLoS One. 2016 Jun 9;11(6):e0157103. doi: 10.1371/journal.pone.0157103 (PMC4900519; doi:10.1371/journal.pone.0157103)
Supplement: S2 Table — (DOCX) [file pone.0157103.s002.docx]

**S2 Table. Accession numbers of HBV datasets used in different analysis**

| C gene n=102  (genotyping) | S gene n=110  (genotyping) | Full genome n=61  (All genotype phylodynamic1991~2010) | Full genome n=45  (Type A phylodynamic1993~2009) |
| --- | --- | --- | --- |
| KC836830 (AH1) | KC836778 (AH1) | D50518 | KC836877 (AH2) |
| KC836831 (AH2) | KC836779 (AH2) | D50519 | KC836878 (AH3) |
| KC836832 (AH3) | KC836780 (AH3) | KC836877 (AH2) | KC836879 (AH22) |
| KC836833 (AH4) | KC836781 (AH4) | KC836878 (AH3) | KC836880 (AH25) |
| KC836834 (AH5) | KC836782 (AH5) | KC836879 (AH22) | KC836881 (AH32) |
| KC836835 (AH6) | KC836783 (AH6) | KC836880 (AH25) | AB205118 |
| KC836836 (AH10) | KC836784 (AH7) | KC836881 (AH32) | AB246338 |
| KC836837 (AH11) | KC836785 (AH9) | AB642092 | AB480041 |
| KC836838 (AH12) | KC836786 (AH10) | AB642093 | AB453979 |
| KC836839 (AH13) | KC836787 (AH11) | AB642094 | AB453980 |
| KC836840 (AH14) | KC836788 (AH12) | AB642095 | AB453981 |
| KC836841 (AH16) | KC836789 (AH13) | AB642096 | AB453982 |
| KC836842 (AH18) | KC836790 (AH14) | AB697490 | AB453983 |
| KC836843 (AH19) | KC836791 (AH15) | AB205118 | AB453984 |
| KC836844 (AH20) | KC836792 (AH16) | AB697506 | AB453985 |
| KC836845 (AH21) | KC836793 (AH18) | AB246338 | AB453986 |
| KC836846 (AH22) | KC836794 (AH19) | AB697507 | AB453987 |
| KC836847 (AH23) | KC836795 (AH20) | AB480041 | AB453988 |
| KC836848 (AH24) | KC836796 (AH21) | AB697509 | AB453989 |
| KC836849 (AH25) | KC836797 (AH22) | AB453979 | AB697512 |
| KC836850 (AH27) | KC836798 (AH23) | AB480038 | AB697511 |
| KC836851 (AH28) | KC836799 (AH24) | AB697504 | AB697509 |
| KC836852 (AH30) | KC836800 (AH25) | AB697501 | AB697508 |
| KC836853 (AH31) | KC836801 (AH27) | AB697489 | AB697507 |
| KC836854 (AH32) | KC836802 (AH28) | AB697512 | AB697506 |
| KC836855 (AH34) | KC836803 (AH29) | AB697511 | AB697505 |
| KC836856 (AH36) | KC836804 (AH30) | AB697488 | AB697504 |
| KC836857 (AH37) | KC836805 (AH31) | AB300367 | AB697503 |
| KC836858 (AH38) | KC836806 (AH32) | AB697505 | AB697501 |
| KC836859 (AH40) | KC836807 (AH33) | AB697503 | AB697489 |
| KC836860 (AH41) | KC836808 (AH34) | AB697491 | AB480038 |
| KC836861 (AH45) | KC836809 (AH35) | AB697508 | AB697488 |
| KC836862 (AH46) | KC836810 (AH36) | AB697496 | AB300367 |
| KC836863 (AH47) | KC836811 (AH37) | AB697497 | AB697491 |
| KC836864 (AH48) | KC836812 (AH38) | AB697499 | AB697496 |
| KC836865 (AH49) | KC836813 (AH40) | AB697487 | AB697497 |
| KC836866 (AH50) | KC836814 (AH41) | AB549213 | AB697498 |
| KC836867 (AH51) | KC836815 (AH42) | AB697492 | AB697499 |
| KC836868 (AH52) | KC836816 (AH43) | AB697495 | AB697487 |
| KC836869 (AH53) | KC836817 (AH44) | AB697493 | AB549213 |
| KC836870 (AH56) | KC836818 (AH45) | AB126580 | AB697492 |
| KC836871 (AH57) | KC836819 (AH46) | AB270536 | AB697495 |
| KC836872 (AH58) | KC836820 (AH47) | AB642100 | AB697493 |
| KC836873 (AH59) | KC836821 (AH48) | AB642099 | AB126580 |
| KC836874 (AH60) | KC836822 (AH49) | AB642097 | AB270536 |
| KC836875 (AH61) | KC836823 (AH50) | AB697510 |  |
| KC836876 (AH62) | KC836824 (AH51) | AB697502 |  |
| AY233288 | KC836825 (AH52) | AB697500 |  |
| AY233280 | KC836826 (AH53) | AB697494 |  |
| AB194950 | KC836827 (AH54) | AB298721 |  |
| D50522 | KC836828 (AH56) | AB298720 |  |
| AB073836 | KC836829 (AH57) | AB205152 |  |
| AB033555 | KC836830 (AH58) | AB640730 |  |
| AB100695 | KC836831 (AH59) | AB299858 |  |
| AB219427 | KC836832 (AH60) | AB642091 |  |
| AB014367 | KC836833 (AH61) | AB205121 |  |
| AB014372 | KC836834 (AH62) | AB205119 |  |
| X75665 | X02763 | AB205120 |  |
| AY233296 | D00330 | AB642098 |  |
| AB078032 | AY123041 |  |  |
| AB048702 | J02203 |  |  |
| AB033558 | X75657 |  |  |
| AB086397 | X69798 |  |  |
| AY090460 | AF160501 |  |  |
| AB036905 | AY090454 |  |  |
| AB036907 | AF241408 |  |  |
| AB036912 | AB048704 |  |  |
| AB036911 | AY233288 |  |  |
| AB036919 | AY233280 |  |  |
| AF223965 | AB194950 |  |  |
| AF223962 | AB073836 |  |  |
| AF223963 | AB033555 |  |  |
| X02763 | AB100695 |  |  |
| D00330 | AB219427 |  |  |
| AY123041 | AB014367 |  |  |
| J02203 | AB014372 |  |  |
| X75657 | X75665 |  |  |
| X75664 | AY233296 |  |  |
| AB091256 | AB078032 |  |  |
| X69798 | AB048702 |  |  |
| AF160501 | AB033558 |  |  |
| AB064310 | X75664 |  |  |
| AB056513 | AB091256 |  |  |
| AB064315 | AB086397 |  |  |
| AB059659 | AB036905 |  |  |
| AB059660 | AB036912 |  |  |
| AB059661 | AB036911 |  |  |
| AY090460 | AB036919 |  |  |
| FJ023661 | AF223965 |  |  |
| FJ023663 | AF223962 |  |  |
| FJ023660 | AF223963 |  |  |
| AF241408 | AB064310 |  |  |
| AB231908 | AB056513 |  |  |
| AF241409 | AB064315 |  |  |
| AB486012 | AB059659 |  |  |
| AB048705 | AB059660 |  |  |
| AB048704 | AB059661 |  |  |
| AJ131571 | AY090460 |  |  |
| AJ131569 | FJ023661 |  |  |
| AJ131572 | FJ023663 |  |  |
| AJ131573 | FJ023660 |  |  |
| AJ131574 | AB231908 |  |  |
|  | AF241409 |  |  |
|  | AB486012 |  |  |
|  | AB048705 |  |  |
|  | AJ131571 |  |  |
|  | AJ131569 |  |  |
|  | AJ131572 |  |  |
|  | AJ131573 |  |  |
|  | AJ131574 |  |  |
